# Supplementary material for: A heritability-based comparison of methods used to cluster 16S rRNA gene sequences into operational taxonomic units
Source: PeerJ. 2016 Aug 30;4:e2341. doi: 10.7717/peerj.2341 (PMC5012273; doi:10.7717/peerj.2341)
Supplement: Supplemental Information 1 — Details of the analysis pipeline used and an outline of each clustering method. [file peerj-04-2341-s001.docx]

**Overview of clustering approaches:**

**UCLUST De Novo** (Edgar 2010)

The UCLUST algorithm developed by Robert Edgar parses a sequence list in the order of the given input (by default in QIIME this order is from most to least abundant). Starting from this first sequence UCLUST aims to generate centroids, represented by a seed or centroid sequence, from these data such that each centroids sequence is less than the % identity threshold similar to all the other centroid sequences, and that all the sequences within the centroid are greater or equal to the % identity threshold in similarity to the seed sequence. % Identity is based on global sequence alignments (considering full read lengths of all sequences in the alignment). Reads are not discarded, as any not matching to an existing centroid will become a new centroid. Identification of % distances, is carried out using a kmer based approach within a algorithm called USEARCH.

In this algorithm, all the experimental reads are broken down into kmers or ‘words’ of a given length. Each unique sequence in the data set is split into all possible words of the chosen length and a list of all words observed in the dataset is recorded. The number of shared kmers between reads is used as an approximate measure of sequence similarity. Once a threshold of word similarity is met, those sequences within the threshold are used in a global alignment and sequences falling within the required % identity threshold are grouped as a cluster.

**UCLUST Reference**

This algorithm is the same as the UCLUST de novo clustering; however instead of taking the experimental reads as input for centroids and for clustered sequences, a reference database of 16S reads is used to generate the centroids and then the experimental sequencing reads are assigned to the reference based centroids. Reads not aligning to reference centroids are discarded.

**UCLUST Open Reference** (Navas-Molina et al. 2013)

The open reference approach is offered as part of the QIIME wrapper. In this approach, the reads are clustered against the reference using UCLUST to generate typical closed reference OTUs. The discarded reads not matching the reference are then subjected to UCLUST de novo clustering. The resultant OTU tables are then merged to generate a complete table with closed and de novo OTUs with no reads discarded.

**VSEARCH Abundance & Distance Based** (Rognes et al. 2016)

VSEARCH is an open source alternative to the commercial USEARCH package (which incorporates UCLUST). The VSEARCH algorithm follows a similar kmer based heuristic approach to generate centroids as USEARCH. However, a VSEARCH implements a Needleman-Wunsch dynamic programming approach to full global alignment distances, where as by deafult USEARCH calculates heuristic distances.

The abundance-based approach differs from the distance based where there is a tie (a sequence is > the % similar to multiple groups). In the abundance-based, it is broken by placing the sequence into the cluster that is most abundant (has the most sequences in the dataset) and in the distance-based by placing it into the cluster whose centroid shares the highest identity.

**SUMACLUST** (Mercier et al. 2013)

SUMACLUST is an open source algorithm that approaches clustering similarly to UCLUST. Sequences are parsed in input order (defaults are from highest to lowest abundance) and used to generate centroids matching % identity criteria. Also considering the abundance ratio between the query sequence and centroid sequence to determine if a new cluster should be made (two highly abundance sequences are more likely to be biologically distinct even is very similar in sequence). Kmer based searching is used to find similar sequences as in other heuristic clustering methods. SUMACLUST implements an alternate % identity distance that is measured as the length of the Longest Common Subsequence in an alignment divided by the shortest alignment in the region.

**Swarm** (Mahé et al. 2014)

Swarm is single-linkage based de novo clustering algorithm that does not utilise a single global identity threshold for clustering. When comparing sequence lengths it uses a kmer based filtering step to remove the requirement for all pair-wise distance caluclations. It then calculates Needleman-Wunsch distances between sequences determined to be similar based on kmer comparisons. The kmer threshold is set by default to only consider sequences differing by 1 nt. This identifies closely related sequences to be clustered. Swarm will start at one sequence and group outwards adding sequences to a cluster then finding sequences closely related to that one and so on. This creates chains of sequences linking together OTUs. OTUs are defined by walking the network of links and determining the abundance patterns across the network. Where drop offs in abundance are identified, chains are broken to delineate OTUs from the complete structure. In this way OTU definitions are dynamic, based on the local structure of the sequence and abundance data at each OTU in the data set.

**Minimum Entropy Decomposition** (A. M. Eren et al. 2014)

Minimum entropy decomposition (MED) is a de novo approach to clustering that does not directly utilise any sequence identity measures. It is based on the oligotyping approach to increasing OTU resolution ( a M. Eren et al. 2014). All sequences in the data set must be aligned if reads are of variable length but can be used directly if they cover the same complete primer region. The number of A,T,C and G bases at each position across all sequences is counted. Shannon entropy, a measure of the nucleotides variability, is then calculated for each position. The assumption is that biological differences will be less randomly distributed than sequencing errors. The Shannon entropy is used as a measure of the distribution of variation at each position. The sequences are then split into smaller groups based on their nucleotide at the highest entropy position. This is carried out recursively on each split group, splitting into further groups by their base at each most variable position until the total entropy values no longer meet predetermined thresholds. If a group falls below a minimum abundance threshold its reads are discarded.

**Overview of OTU Clustering Workflow**

Pad Fasta Lengths Using Oligotyping Tool

MED

De-replicated

Sequences

Oligotyping

Decomposition

SUMACLUST

SWARM

Abundance

98%

Abundance

99%

Abundance

97%

Distance

Based

97%

Abundance

Based

VSEARCH

(USEARCH61 as alias)

SWARM

SUMACLUST

De Novo

Closed Reference

Open Reference

UCLUST in QIIME

QIIME

VSEARCH De-replication

Complete Fasta After QC

Select Complete Twin Pairs

Remove All Sequences with Ambiguous Bases and Not of Length 252 or 253nt

Concatenate Sample Files

Remove Chimeric Sequences

De Novo Chimera Checking Using USEARCH61 in QIIME

Split Sequencing By Sample

Fastq Join Paired End Read with 200nt Overlap

Complete MiSeq Sequencing >2800 Samples

**Commands to remove chimeric sequences & trim data (run on individual FASTA files for each sample):**

**Identify chimeras (QIIME):**

identify_chimeric_seqs.py -i sample.fasta -m usearch61 --suppress_usearch61_ref --usearch61_xn 7 -o sample.out

**Concatenate the resultant chimera files into one file then remove chimeras from full data FASTA using (QIIME):**

filter_fasta.py -f full.fasta -o no_chimeras.fasta -s concat_chimeras.txt –n

**Trim to length and remove any with ambiguous bases (Mothur):**

trim.seqs(fasta=no_chimeras.fasta, maxambig=0, minlength=252, maxlength=253)

The resultant file was then used as the input.fasta in the steps below.

**Commands to cluster OTUs for each method:**

QIIME commands were run using QIIME version 1.9.0, where VSEARCH was required USEARCH61 was run as a method within QIIME and an alias used to direct USEARCH61 calls to the VSEARCH version 1.9.3 (Linux) executable.

VSEARCH de-replication was carried out directly using VSEARCH following a workflow provided by Greg Caporaso at <https://gist.github.com/gregcaporaso/f3c042e5eb806349fa18> (last accessed 20th April 2016).

Minimum Entropy Decomposition (MED) was run using the decompose command as part of the oliogtyping pipeline, details of which can be found here: <http://merenlab.org/projects/oligotyping/>

Picking parameters either matched those of the He et al. comparison (He et al. 2015) where applicable or used default values. Where a reference was required Greengenes version 13_8 (DeSantis et al. 2006).

**UCLUST DE NOVO:**

pick_de_novo_otus.py -i input.fasta -o uclust_denovo/ -p uclustdenovo.params.txt

uclustdenovo.params.txt:

pick_otus:otu_picking_method uclust

pick_otus:max_accepts 16

pick_otus:max_rejects 64

pick_otus:enable_rev_strand_match True

**UCLUST CLOSED REFERENCE:**

pick_closed_reference_otus.py -i input.fasta -o uclust_closed_gg_13_8/ -r gg_13_8_otus/rep_set/97_otus.fasta –p closedref.params.uclust.txt

closedref.params.uclust.txt:

pick_otus:otu_picking_method uclust_ref

pick_otus:max_accepts 16

pick_otus:max_rejects 64

pick_otus:enable_rev_strand_match True

pick_otus:minlen 30

**UCLUST OPEN REFERENCE:**

pick_open_reference_otus.py -i input.fasta -o uclust_open_gg_13_8/ -m uclust -r gg_13_8_otus/rep_set/97_otus.fasta -s 1 -p openref.params.uclust.txt --min_otu_size 1 --prefilter_percent_id 0.0

openref.params.uclust.txt:

pick_otus:max_accepts 16

pick_otus:max_rejects 64

pick_otus:enable_rev_strand_match True

**VSEARCH AGC 97, 98 and 99%:**

pick_de_novo_otus.py -i input.fasta -o vsearch_AGC_**% threshold**/ -p agc.params.**% Threshold**.txt

agc.params files:

pick_otus:otu_picking_method usearch61

pick_otus:sizeorder True

pick_otus:max_accepts 16

pick_otus:max_rejects 64

pick_otus:enable_rev_strand_match True

pick_otus:minlen 30

pick_otus:similarity 0.97**0.98**0.99

**VSEARCH DGC 97%:**

pick_de_novo_otus.py -i input.fasta -o vsearch_DGC_97/ -p dgc.params.txt

dgc.params.txt:

pick_otus:otu_picking_method usearch61

pick_otus:max_accepts 16

pick_otus:max_rejects 64

pick_otus:enable_rev_strand_match True

pick_otus:minlen 30

**SUMACLUST:**

pick_de_novo_otus.py -i input.fasta -o sumaclust_denovo/ -p $ sumaclust.params.txt

sumaclust.params.txt:

pick_otus:otu_picking_method sumaclust

pick_otus:threads 16

**SWARM:**

pick_de_novo_otus.py -i input.fasta -o swarm_denovo/ -p swarm.params.txt

swarm.params.txt:

pick_otus:otu_picking_method swarm

pick_otus:threads 16

**VSEARCH DE-REPLICATION:**

vsearch --derep_input.fasta --output vsearch_dereplicated/vsearch_dereplicated_repset.fna --uc vsearch_dereplicated/vsearch_dereplicated.uc --relabel_sha1 --relabel_keep

biom from-uc -i vsearch_dereplicated/vsearch_dereplicated.uc -o vsearch_dereplicated/vsearch_dereplicated_biom.biom --rep-set-fp vsearch_dereplicated/vsearch_dereplicated_repset.fna

**MED:**

o-pad-with-gaps input.fasta -o input_padded.fasta

decompose –i input_padded.fasta –o med/

**Heritability Calculation**

Total OTU counts were taken from complete OTU tables (biom summarize-table) then tables subset to OTUs found in at least 50% of individuals. This enabled the smaller tables to be converted into text formats to load in R whilst retaining complete count information, which was used to convert counts to relative abundances.

R Heritability Script is based on the OpenMX path based ACE modelling example (<http://openmx.psyc.virginia.edu/docs/OpenMx/2.5.1/GeneticEpi_Path.html> accessed last on 30/6/2016).

Heritability R script:

#packages for compostional transforms, lme4 and for open mx

library(OpenMx)

library(lme4)

library(data.table)

library(methods)

library(car)

#read in argument from command line

args = commandArgs(trailingOnly=TRUE)

#otu table for otus in >= 50% samples

otutable=args[1]

#output directory

out=args[2]

#path to OTU counts for samples in complete (all OTUs not just >50%) OTU table

summarypath=args[3]

#total number of OTUs observed in the compelete set

totobs=as.numeric(args[4])

#read in OTU table

bacteria=fread(otutable,header=T,sep="\t")

bacteria=data.frame(bacteria)

rownames(bacteria)=bacteria[,1]

bacteria=bacteria[,-1]

#transform data to abundances considering total number of OTUs and adding a pseduo count of 1 for all OTUs

bacteria1=bacteria

summary=read.table(summarypath)

summary=summary[match(colnames(bacteria1),summary[,1]),]

for(i in 1:nrow(summary)){

summary[i,2]=summary[i,2]+totobs

}

#summary now represents the total number of OTUs in each sample in the complete OTU table, plus a count of 1 on every OTU

#add a count of 1 to the zeros on the OTU table

bacteria1=bacteria1+1

#convert to relative abundances

for(i in 1:ncol(bacteria1)){

bacteria1[,i]=bacteria1[,i]/summary[i,2]

}

#clrb used from now on to hold otu counts

clrb=bacteria1

#read in mapping file

sampdat=read.csv("twins_mapping_file.csv",header=T)

#read in file summarising the read depth in each sample

sampseqcount=read.table("twins_seq_count.txt",header=T,sep='\t')

sampdat=merge(sampdat,sampseqcount,by="X.SampleID")

#create box cox transformed residuals after controlling for covariates

#subset the data to the twins in the study from the mapping and OTU tables make all the files the same order

clrb=t(clrb)

clrb=clrb[which(rownames(clrb)%in%sampdat$X.SampleID),]

sampdat=sampdat[which(sampdat$X.SampleID%in%rownames(clrb)),]

sampdat=sampdat[match(rownames(clrb),sampdat$X.SampleID),]

#clrbresids will hold the residuals

clrbresids=clrb

#scale covariates from mapping to same ranges

#age

sage=scale(sampdat$age_at_sample,center = T)

#sequencing depth

sscount=scale(sampdat$SeqCount,center=T)

gender=as.factor(sampdat$i.gender)

#sequencing run

seqrun=as.factor(sampdat$p.SequencingRun)

#who loaded and extracted the data

loaded=as.factor(sampdat$e.Loadedby)

extracted=as.factor(sampdat$e.Extractedby)

#sample collected in person or from postal kit

collect=as.factor(sampdat$s.CollectionMethod)

#generate box cox transformed residuals otu by otu

for(i in 1:ncol(clrb)){

otu=clrb[,i]

mod=lm(otu~gender+sage+seqrun+sscount+loaded+extracted+collect)

#estimate box cox lamda and normalise

a=powerTransform(otu)

mod=lm(bcPower(otu,a$roundlam)~gender+sage+seqrun+sscount+loaded+extracted+collect)

resids=summary(mod)$residuals

#overwrite the column with residuals

clrbresids[,i]=resids

}

#create twin format for the openMX ACE script

fam=c()

twin1=c()

twin2=c()

zyg=c()

for(i in unique(sampdat$i.FamilyID)){

dat=sampdat[which(sampdat$i.FamilyID==i),]

fam=c(fam,i)

twin1=c(twin1,which(sampdat$X.SampleID==dat$X.SampleID[1]))

twin2=c(twin2,which(sampdat$X.SampleID==dat$X.SampleID[2]))

zyg=c(zyg,as.character(dat$i.zygosity[1]))

}

t1dz=twin1[which(zyg=="DZ")]

t2dz=twin2[which(zyg=="DZ")]

t1mz=twin1[which(zyg=="MZ")]

t2mz=twin2[which(zyg=="MZ")]

fdz=fam[which(zyg=="DZ")]

fmz=fam[which(zyg=="MZ")]

#vectors to hold estimates and CI

a=c()

al=c()

au=c()

c=c()

cl=c()

cu=c()

e=c()

el=c()

eu=c()

#calculate hertiabilties for each OTU reformatting data

for(i in 1:ncol(clrbresids)){

otudat=clrbresids[,i]

otunamer=colnames(clrbresids)[i]

#get data for pairs

otu1dz=otudat[t1dz]

otu2dz=otudat[t2dz]

otu1mz=otudat[t1mz]

otu2mz=otudat[t2mz]

#group

mzData=cbind(otu1mz,otu2mz)

dzData=cbind(otu1dz,otu2dz)

#colnames to sel vars

selVars=c("otut1","otut2")

colnames(mzData)=selVars

colnames(dzData)=selVars

#define latent ace variables

aceVars=c("A1","C1","E1","A2","C2","E2")

#means and covs

mzmeans=colMeans(mzData,na.rm=TRUE)

dzmeans=colMeans(dzData,na.rm=TRUE)

mzcov=cov(mzData,use="complete")

dzcov=cov(dzData,use="complete")

# Path objects for Multiple Groups

manifestVars=selVars

latentVars=aceVars

# variances of latent variables

latVariances <- mxPath( from=aceVars, arrows=2,

free=FALSE, values=1 )

# means of latent variables

latMeans <- mxPath( from="one", to=aceVars, arrows=1,

free=FALSE, values=0 )

# means of observed variables

obsMeans <- mxPath( from="one", to=selVars, arrows=1,

free=TRUE, values=0, labels="mean" )

# path coefficients for twin 1

pathAceT1 <- mxPath( from=c("A1","C1","E1"), to="otut1", arrows=1,

free=TRUE, values=.33, label=c("a","c","e") )

# path coefficients for twin 2

pathAceT2 <- mxPath( from=c("A2","C2","E2"), to="otut2", arrows=1,

free=TRUE, values=.33, label=c("a","c","e") )

# covariance between C1 & C2

covC1C2 <- mxPath( from="C1", to="C2", arrows=2,

free=FALSE, values=1 )

# covariance between A1 & A2 in MZ twins

covA1A2_MZ <- mxPath( from="A1", to="A2", arrows=2,

free=FALSE, values=1 )

# covariance between A1 & A2 in DZ twins

covA1A2_DZ <- mxPath( from="A1", to="A2", arrows=2,

free=FALSE, values=.5 )

# Data objects for Multiple Groups

dataMZ <- mxData( observed=mzData, type="raw" )

dataDZ <- mxData( observed=dzData, type="raw" )

# Combine Groups

paths <- list( latVariances, latMeans, obsMeans,

pathAceT1, pathAceT2, covC1C2 )

modelMZ <- mxModel(model="MZ", type="RAM", manifestVars=selVars,

latentVars=aceVars, paths, covA1A2_MZ, dataMZ )

modelDZ <- mxModel(model="DZ", type="RAM", manifestVars=selVars,

latentVars=aceVars, paths, covA1A2_DZ, dataDZ )

minus2ll <- mxAlgebra( expression=MZ.fitfunction + DZ.fitfunction,

name="minus2loglikelihood" )

obj <- mxFitFunctionAlgebra( "minus2loglikelihood" )

estVA <- mxAlgebra(expression=a*a,name="a2")

estVC <- mxAlgebra(expression=c*c,name="c2")

estVE <- mxAlgebra(expression=e*e,name="e2")

estVP <- mxAlgebra(expression=a2+c2+e2, name="V")

estPropVA <- mxAlgebra(expression=a2/V, name="A")

estPropVC <- mxAlgebra(expression=c2/V, name="C")

estPropVE <- mxAlgebra(expression=e2/V, name="E")

modelACE <- mxModel(model="ACE", modelMZ, modelDZ, minus2ll,obj, estVA,estVC,estVE,estVP,estPropVA,estPropVC,estPropVE,mxCI(c("A","C","E")))

# Run Model

fitACE <- mxRun(modelACE,intervals=TRUE)

a2l=fitACE$output$confidenceIntervals[1,1]

a2=fitACE$output$confidenceIntervals[1,2]

a2u=fitACE$output$confidenceIntervals[1,3]

c2l=fitACE$output$confidenceIntervals[2,1]

c2=fitACE$output$confidenceIntervals[2,2]

c2u=fitACE$output$confidenceIntervals[2,3]

e2l=fitACE$output$confidenceIntervals[3,1]

e2=fitACE$output$confidenceIntervals[3,2]

e2u=fitACE$output$confidenceIntervals[3,3]

a=c(a,a2)

al=c(al,a2l)

au=c(au,a2u)

c=c(c,c2)

cl=c(cl,c2l)

cu=c(cu,c2u)

e=c(e,e2)

el=c(el,e2l)

eu=c(eu,e2u)

}

#combine results into a data frame

res=data.frame(colnames(clrbresids),al,a,au,cl,c,cu,el,e,eu)

**References:**

DeSantis, T.Z. et al., 2006. Greengenes, a Chimera-Checked 16S rRNA Gene Database and Workbench Compatible with ARB. *Applied and Environmental Microbiology*, 72(7), pp.5069–5072. Available at: http://aem.asm.org/cgi/doi/10.1128/AEM.03006-05.

Edgar, R.C., 2010. Search and clustering orders of magnitude faster than BLAST. *Bioinformatics*, 26(19), pp.2460–2461. Available at: http://bioinformatics.oxfordjournals.org/cgi/doi/10.1093/bioinformatics/btq461.

Eren, a M. et al., 2014. Oligotyping analysis of the human oral microbiome. *Proceedings of the National Academy of Sciences*, 111(28), pp.E2875–E2884. Available at: http://www.ncbi.nlm.nih.gov/pubmed/24965363.

Eren, A.M. et al., 2014. Minimum entropy decomposition: Unsupervised oligotyping for sensitive partitioning of high-throughput marker gene sequences. *The ISME Journal*, 9(4), pp.968–979. Available at: http://www.nature.com/doifinder/10.1038/ismej.2014.195.

He, Y. et al., 2015. Stability of operational taxonomic units: an important but neglected property for analyzing microbial diversity. *Microbiome*, 3(1), p.20. Available at: http://www.microbiomejournal.com/content/3/1/20.

Mahé, F. et al., 2014. Swarm: robust and fast clustering method for amplicon-based studies. *PeerJ*, 2, p.e593. Available at: http://dx.doi.org/10.7717/peerj.593.

Mercier, C. et al., 2013. SUMATRA and SUMACLUST : fast and exact comparison and clustering of sequences. *Abstract In: SeqBio 25-26th Nov 2013*, p.27. Available at: http://doi.wiley.com/10.1002/ejoc.201200111.

Navas-Molina, J.A. et al., 2013. Advancing Our Understanding of the Human Microbiome Using QIIME. In *Microbial Metagenomics, Metatranscriptomics, and Metaproteomics*. Elsevier Inc., pp. 371–444. Available at: http://linkinghub.elsevier.com/retrieve/pii/B9780124078635000198.

Rognes, T. et al., 2016. VSEARCH. Available at: https://github.com/torognes/vsearch.
